# Printable Resin Modified by Grafted Silver Nanoparticles for Preparation of Antifouling Microstructures with Antibacterial Effect

**DOI:** 10.3390/polym13213838

**Published:** 2021-11-06

**Authors:** Hazem Idriss, Roman Elashnikov, Silvie Rimpelová, Barbora Vokatá, Petr Haušild, Zdeňka Kolská, Oleksiy Lyukatov, Václav Švorčík

**Affiliations:** 1 Department of Solid State Engineering, University of Chemistry and Technology, Prague, Technická 3, 166 28 Prague, Czech Republic; elashnir@vscht.cz (R.E.); luytakoo@vscht.cz (O.L.); vaclav.svorcik@vscht.cz (V.Š.); 2 Department of Biochemistry and Microbiology, University of Chemistry and Technology, Prague, Technická 3, 166 28 Prague, Czech Republic; barbora.vokata@vscht.cz; 3 Department of Materials, Faculty of Nuclear Sciences and Physical Engineering, Czech Technical University, 120 00 Prague, Czech Republic; petr.hausild@fjfi.cvut.cz; 4 Materials Centre, Faculty of Science, J. E. Purkyně University, 400 96 Ustí nad Labem, Czech Republic; zdenka.kolska@seznam.cz

**Keywords:** silver nanoparticles, diazonium salt, antibacterial activity, biomaterial, CAD/CAM, antifouling properties

## Abstract

The usage of three-dimensional (3D) printed materials in many bioapplications has been one of the fastest-growing sectors in the nanobiomaterial industry in the last couple of years. In this work, we present a chemical approach for grafting silver nanoparticles (AgNPs) into a resin matrix, which is convenient for 3D printing. In this way, the samples can be prepared and are able to release silver ions (Ag^+^) with excellent antibacterial effect against bacterial strains of *E. coli* and *S. epidermidis*. By the proposed process, the AgNPs are perfectly mixed and involved in the polymerization process and their distribution in the matrix is homogenous. It was also demonstrated that this approach does not affect the printing resolution and the resin is therefore suitable for the construction of microstructures enabling controlled silver ion release and antifouling properties. At the same time the physical properties of the material, such as viscosity and elasticity modulus are preserved. The described approach can be used for the fabrication of facile, low-cost 3D printed resin with antifouling-antibacterial properties with the possibility to control the release of Ag^+^ through microstructuring.

## 1. Introduction

Since the production of the first polymer in the late 19th century, the number of polymer applications increases every day, due to their relatively low cost, excellent physical properties, and high biocompatibility. The high ductility of many monomers clears the way for using them in 3D printing techniques such as selectively deposited layer (SDL) and other computer-aided design/computer-aided manufacturing (CAD/CAM) methods. Also, additional techniques providing the higher resolution required for specific needs and highly demanding bioapplications such as release and delivery of antibiotics or their alternatives (for example, silver or iron oxide nanoparticles) or achieving antifouling properties can be mentioned. These aims are even more urgent concerning the ever-increasing bacterial resistance against commonly used antibiotics. The use of silver nanoparticles (AgNPs) is still an important approach in producing a variety of bioapplicable materials with antimicrobial properties [1,2], suitable for wound healing [3], or drug delivery [4], Smart antimicrobial materials [5], or substrates with silver nanoparticles [6] with on-demand drug release are highly desired for biomedical applications. One very good method for their preparation is that proposed by Gomez-Carretero et al. [7]. They used AgNPs as a coating on different polymers such as polystyrene sulfonate with covalent binding through an electro-enhanced approach. Materials prepared in this way exhibit good antimicrobial effects and chemical and mechanical stability, but like many others, they are not suitable for sample manufacturing by 3D printing, which is the only one with the required resolution of the prepared structures. In another approach, the required samples were soaked or immersed in an active solution containing AgNPs or Ag ions. In this simple way, the samples acquired antibacterial properties from the embedded AgNPs [8,9]. This method might be particularly promising due to its simplicity and environment-friendly character. On the other hand, the chemical stability of the attached functional group has to be further examined if the products should be used in low pH environments (for example, in the case of inflammation treatment). The embedding of AgNPs into the material structure is one of the main methods by which the materials achieve antibiotic characteristics. Recently this technique has been significantly improved [10,11,12]. The new techniques are rapidly applicable to a variety of materials including polymers, oligomers, composites, and alloys and they enable one to control the AgNP concentration. In this work, we present an alternative approach to preparing an antibacterial/antifouling resin for bioapplications. Firstly, the AgNPs were grafted in situ with diazonium salt (ADT-CH = CH_2_), then mixed with methyl methacrylate (MMA) monomer-based resin and polymerized under violet light at a wavelength of 405 nm. The samples were characterized by many methods. Surface morphology was examined by confocal microscopy, the nanoparticles were observed by transmission electron microscopy (TEM) and the viscosity of the liquid resin was studied by viscosimetry. Also, the mechanical properties were evaluated and the chemical composition was determined by infrared spectroscopy (IR), scanning electron microscopy (SEM) with energy-dispersive X-ray spectroscopy (EDX), and UV-Vis spectrophotometry (UV-Vis). These spectra of deionized water in which the samples were soaked also confirmed that silver is released in the Ag^+^ (Ag ion).form. The prepared resin showed significant antibacterial and antifouling potential. Since the resin preserved its original physical properties, it can be printed by an SDL 3D printer and used in various applications, in which these highly demanding properties are needed.

## 2. Materials and Methods

### 2.1. Materials

The material used was resin-based MMA, which is compatible with the SDL resin printer. As a resin filler, the AgNPs in the form of crystals (#85131) were used. *p*-Toluenesulfonic acid monohydrate (P-TsOH) and *tert*-butyl nitrite (tBuONO, ADT-CH = CH_2_) were used for grafting. The AgNPs and all chemicals were purchased from Sigma Aldrich (St. Louis, MO, USA). To prepare the samples (square-shaped L = 10 mm, h = 2 mm), we used a high-resolution laser light SDL 3D printer Aurora F1 (Aurora Group, Tapei City, Taiwan) main movable platform which was modified in the light of previous works [13,14] by adding a laser displacement sensor, an electromagnetic actuator, and a feedback controller and also a violet laser light with the wavelength of 405 nm.

### 2.2. Silver Nanoparticles Preparation

**Figure 1:**
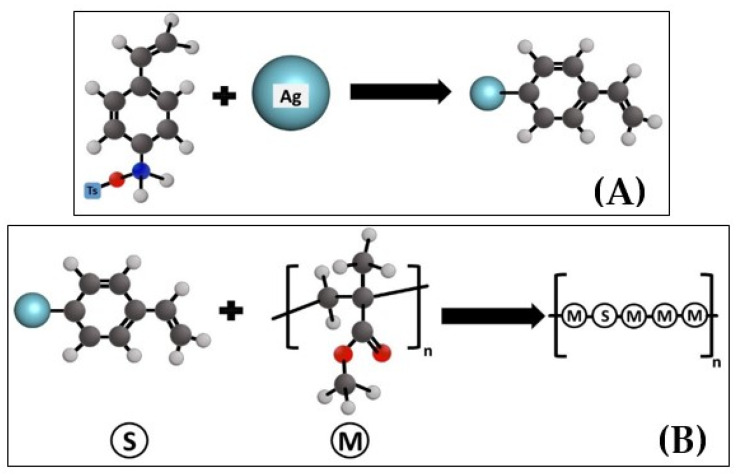
Schematic representation of AgNPs grafting with 4-aminostyrene (**A**); polymerization of the monomers mixed with modified AgNPs (**B**). Ts = *p*-toluenesulfonic acid monohydrate; S - styrene, M - monomer.

The AgNPs were prepared and grafted with diazonium salt [15] according to the following procedure. Firstly, to graft 0.1 g of AgNPs, 0.285 g of *p*-TsOH was dissolved in 5 mL of CH_3_COOH. After the complete dissolution of 0.18 g of tBuONO, 0.12 g of ADT-CH = CH_2_ was added to the solution. All components were mixed for 1 h. The above-measured amount of ungrafted AgNPs was added to the solution. It was mixed again for an additional 1 h (see Figure 1), a process that separates and grafts the AgNPs. Then, it was washed (5×) with 5 mL of methanol (MeOH, 99.97%) with centrifuging after every wash for 15 min. at a speed of 7500 rpm until it became colorless and finally washed with 5 mL of acetone (99.98%) and centrifuged again. All of these steps were performed with a magnetic stirrer at a temperature of 25 °C. The resulting powder is a separated grafted AgNPs.

The resin was used without any further modification. Three different groups were prepared according to the concentrations of grafted AgNPs in the liquid resin according to the weight used starting from 5, 10, and 15 wt.% and one control group without AgNPs (pristine samples).

After printing the samples, they were soaked in deionized water (5 mL for each sample) and divided into five groups according to the time of soaking; 24, 48, 72, 96, and 168 h. The water was retained and later used for: (i) identification of the form of the released Ag, (ii) determination of the Ag concentration in the water according to the time of soaking/concentration in the solid samples, and (iii) performing antibacterial tests.

### 2.3. Characterization Methods

#### 2.3.1. Physical and Chemical Characterization

Physical characterization of the materials was performed on both the pre-solidified resin (liquid) and the resin after solidification. Testing of the liquid resin is important due to the 3D printer’s ability, to “flow” and pipe the resin and the ability to solidify it with the same resolution. Therefore, the viscosity of the resin-modified by different AgNPs concentrations (in liquid phase) was examined by a Brookfield Rheometer DV3TLVTJO (AMETEK, Wilmington, MA, USA).

The concentration of the grafted AgNPs in the liquid resin was determined from the absorbance spectra of the samples taken by a UV-Vis spectrometer (Lambda 25, PerkinElmer, Chorley, UK). The spectrum from the pristine sample served as a reference and the concentration in the samples containing AgNPs was calculated as reported before [16] using the equation: C = A/(L × ε), in which A is absorption at λ_max_, L is the path length, which is in our case the length of the side of the cuvette’s base, and ε is the extinction coefficient, which can also be measured using Lambert-Beer’s law at the maximum absorption of the grafted AgNPs [17].

The morphology of the surface of the solid grafted samples with different AgNP concentrations was studied by confocal microscopy (Olympus OLS 3100, Olympus, Tokyo, Japan), used on the micron scale.

The mechanical properties of four types of solid samples were determined: a pristine one and ones with 15% of grafted AgNPs after being soaked in deionized water for 1 week and 1 month (to understand the effect of Ag ions release on mechanical properties of the material). Nanoindentation tests were carried out using an NHT2 tester (Anton Paar, Graz, Austria) equipped with a Berkovich diamond indentor using instrumented indentation technique [18]. The maximum load was P_max_ = 50 mN, the loading and unloading time was 30 s, the hold period at the maximum load was 10 s. The acquired data (force-depth of penetration data record) were evaluated to obtain hardness HIT and plane strain elastic modulus E* according to the ISO 14577 standard by the Oliver-Pharr method [19].

The chemical changes on AgNPs after the grafting and the chemical interaction between the grafted AgNPs and the resin were investigated (3000 scans and 4 cm^−1^ resolution) by Fourier transform infrared (FTIR) spectrometry with a Nicolet 6700 spectrometer (Thermo Scientific, Waltham, MA, USA) equipped with a SMART ATR accessory. The spectra of pristine samples (non-modified, without AgNPs) and samples grafted with AgNPs (modified resins) were gathered. Special attention was paid to the new peaks appearing in the spectra of the grafted AgNPs samples. The AgNPs grafting is supposed to result in several transformations, which can be detected or interpreted from the FTIR spectra (for instance the occurrence of 1,4-disubstituted benzenes [780–860 cm^−1^] and [680–710 cm^−1^], vinyl- group signal C=C in the range of [1465–1640 cm^−1^], aromatic ring = C–H and C=C vibrations between [3000–3105 cm^−1^], and [1590–1625 cm^−1^], respectively, and aromatic alkene bonds (C–H, [1010–1180 cm^−1^]) [20].

The distribution of the AgNPs inside the samples was studied by the SEM-EDX technique. The sample was cut longitudinally, which might give the average distribution of the particles. After that, the samples were sputtered with a gold layer with a thickness of 5 nm to make the surface conductive. The samples were examined on both the inner and outer surfaces at 10 different positions.

The second test was aimed at determining the form in which the silver ions are released into the environment. For that purpose, UV-Vis spectra were gathered for two aqueous samples, prepared as follows: firstly 0.1 mg of non-grafted silver was dissolved in deionized water. The solution was centrifuged for 15 min. at 7000 rpm. The solid particles were separated from the liquid and the spectrometer was used to gather the absorption spectrum of the liquid part. The second sample was deionized water in which the solid-modified resin was soaked for one month. Both liquids were studied, considering deionized water as the zero line.

The concentration of Ag in solution was determined by two methods: (i) addition (titration) with HNO_3_ and (ii) analysis by the AAS flame atomization technique. Both of them gave convergent results with an error of 0–5%.

#### 2.3.2. Material Functionality and Its Antibacterial Properties

The functionality of the samples was studied in two main directions. First, the antibacterial effect, i.e., the ability of the samples to kill/inhibit the growth and/or multiplication of bacteria (bactericidal and bacteriostatic effect). The antibacterial properties of the samples were evaluated against two environmental bacterial strains: a Gram-negative strain of *Escherichia coli* (*E. coli*, DBM 3138) and a Gram-positive strain of *Staphylococcus epidermidis* (*S. epidermidis*, DBM 2124). Both strains were obtained from the collection of microorganisms at the Department of Biochemistry and Microbiology of the University of Chemistry and Technology (Prague, Czech Republic). The method used to evaluate the antibacterial properties was the drop plate method [21,22,23,24]. The Luria-Bertani medium was inoculated with one colony-forming unit (CFU) of each bacterial strain and cultivated in an orbital shaker at 37 °C for 16 h. Then, the inocula were serially diluted in sterile phosphate-buffered saline (PBS, pH 7.4). The final concentration of bacteria in the solution was 1 × 10^4^ per mL.

The samples were prepared in triplicate. One hundred μL of the liquid in which samples were soaked, was added to 2 mL of the final bacterial suspension. Then 25 μL aliquots from each sample were gently vortexed and placed on pre-dried agar plates (*E. coli* on LB agar plates, *S. epidermidis* on PCA agar plates) in five technical replicates and incubated at 37 °C for 24 h. Then, the number of CFU of each strain was then counted. The experiments were performed under sterile conditions.

Another property that plays a key role in the total antibacterial effect is to make the surface more “repellent” to bacteria, i.e., preventing the bacteria from adhering to the surface of the samples and also removing dead bacteria “debris”. This can be especially important if the residues of the dead bacteria might be used by other species to form a biofilm and adhere better to the surface. Also, if long-term usage is intended, the bacteria could exhaust the AgNPs in the material. In such cases, the material’s surface should not support bacterial growth since it might cause inflammation, rejection by the body, and eventually failure of the biomaterial application. To prevent that, we printed additional microstructures on the surface of the samples from the corresponding material (resin with or without AgNPs) as was suggested by Kennedy and Vasudevan [25,26] which is repeated on a large shape (23 × 23 × 21 μm^3^) and a spacing of 5 μm.

Next, dynamic antibacterial tests were performed at a flow rate of 2.5 mL·min^−1^. For this purpose, the samples were placed in the flow of bacterial suspension with the optical density (OD) of 1 for 1 h, separately, using a precise peristaltic pump BT100-2. (Halma, Amersham, city, UK) After 1 h, the samples were fixed by the solution of 2% formaldehyde (methanol-free, 16% stock solution, Thermo-Fisher Scientific) with 2.5% glutaraldehyde (EM grade) in phosphate buffer saline (pH = 7.4) for 2 h at ambient temperature. After that, the samples were dehydrated by increasing the concentration of ethanol (50, 60, 70, 80, 90, and 2 × absolute) with every step taking 10 min, at the end the samples were dried overnight at a temperature of 37 °C [27]. Finally, the dehydrated samples were sputtered by a 5-nm platinum layer.

To study the sample structures, we evaluated the SEM images of all samples (each in 10 different positions). The search for bacteria was performed for heights ranging from 0–21 μm. The bacteria were counted for all of the samples and one-way analysis of variance ANOVA was performed to study the significance of means’ differences of the samples (within groups and between groups) by using the OriginLab Pro 2018b (OriginLab Corporation, Northampton, MA, USA) software.

Another feature of such material is the possibility to control the release of Ag+. This can be achieved by increasing the total square surface (or volume) of the samples, but due to the limited space available in many cases, this was not applicable. To overcome this problem, microstructures similar to the ones used when performing the antifouling test were printed, with a variable microstructure height of 21, 42, and 84 μm (while the other dimensions remained unchanged). As a result, the total surface area was increased by ∑ A× X, where ∑A is the sum of all sides of the (added) shape (excluding the upper one) and X is the number of printed shapes.

## 3. Results and Discussion

**Figure 2:**
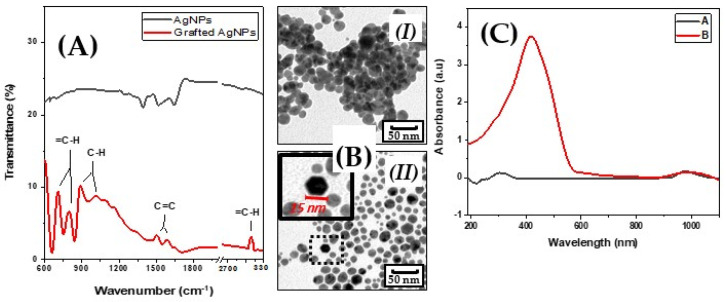
Confirmation of successful modification of AgNPs by IR spectroscopy (**A**); morphology of silver nanoparticles (AgNPs) determined by TEM (**I**) before grafting and (**II**) after grafting (**B**); absorbance spectrum of aqueous silver solution (**I**) and of deionized water in which the samples were soaked (**C**).

Grafting AgNPs on MMA with 4-aminostyrene as aforementioned formed a silver-styrene composite. These grafted AgNPs were added to the monomer liquid (schematically represented in Figure 1). We assume that the grafted AgNPs inside the structure of resin disintegrate releasing silver ions Ag^+^, which have an antibacterial effect. The successful grafting of the AgNPs was confirmed by IR spectroscopy, which showed new peaks appearing at 706 and 800 cm^−1^ for 1,4-disubstituted benzenes, 890, and 1010 cm^−1^ for the C–H alkyl bond, 1510 cm^−1^ for the vinyl-group C=C, and 1590 cm^−1^ for the aromatic group C=C and finally 3090 cm^−1^ for the aromatic bond =C–H (Figure 2A).

The images obtained from TEM analysis showed different behavior of the nanoparticles. The non-grafted particles were aggregated in clusters and it was impossible to separate them even by using ultrasound (Figure 2B (I)). After their grafting, the clusters disappeared and the particles became separated. This process can be connected with the changes in the surface energy or alterations of the electrostatic energy that occurred after the modification (Figure 2B (II)).

The UV-Vis spectra of deionized water, in which the samples were soaked, and the solution of non-grafted silver was measured to detect the form of the released Ag, are presented in Figure 2C. The obtained results show that the silver was released in Ag^+^ form.

**Figure 3:**
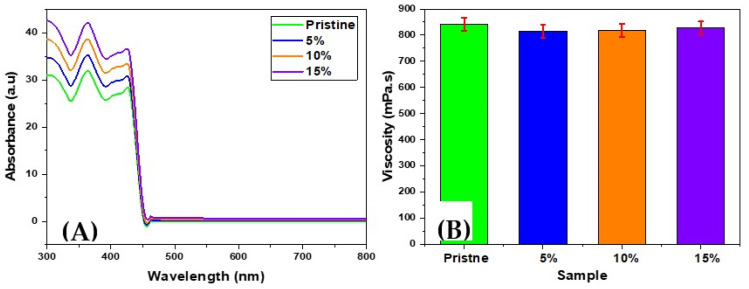
Absorbance spectra of different concentrations of the AgNPs-modified resin (**A**); viscosity test of the modified samples (liquid) in comparison with the pristine samples (**B**).

The concentration of the grafted AgNPs inside the resin was determined using UV-Vis spectroscopy. The spectra presented in Figure 3A show a linear relationship between intended and measured concentrations. Both agree with the experimental error (see Figure 3A).

The tests of the resin viscosity in the liquid phase showed convergent results for all modified samples and the pristine samples as well. These results show that the flowability and the ductility of the liquid are with minor changes preserved and the printer will be able to pump the resin and construct a high-resolution structure as if it was pristine (Figure 3B).

**Figure 4:**
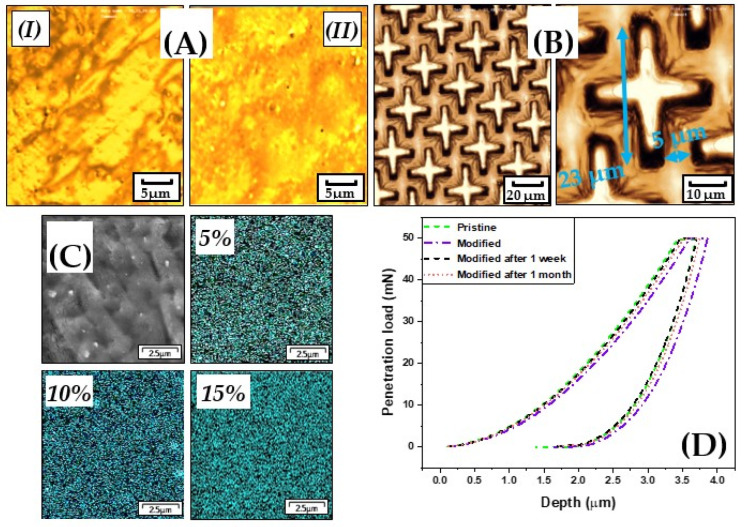
Surface morphology of pristine (**I**) and modified samples (**II**) determined by confocal microscopy (**A**); printed structures on the surface (**B**); overview of the surface of the modified samples measured by SEM and mapping distribution of AgNPs in different concentrations inside the samples acquired by EDX (**C**); load-depth of penetration instrumented indentation test data record of pristine samples, modified samples, and modified samples after releasing silver ions for one week and one month (**D**).

After printing or solidifying the resin, the surface morphology was examined by confocal microscopy to track the surface changes or any possibly vast surface defects. No significant changes or defects on the surface were found as is clear from Figure 4A, so it was possible to print the designed microstructures with high accuracy and with minimal defects, which can be seen in Figure 4B.

From the SEM analysis, it is apparent that the AgNPs (Figure 4C) exhibited a homogenous distribution at any examined spot. This is very important since the antibacterial effect would be the same for any side or sector of the sample and even, as it is clear, also AgNPs distribution did not depend on their concentration.

The results of the indentation measurements are summarized in Table 1 and the load-depth of penetration curves are shown in Figure 4D. It can be seen that the modification by AgNPs and subsequent soaking in deionized water (for 1 week and 1 month) led to only a slight variation in hardness and/or in elastic modulus, i.e., the mechanical properties of the modified resin were not significantly affected by the modification procedure.

**Figure 5:**
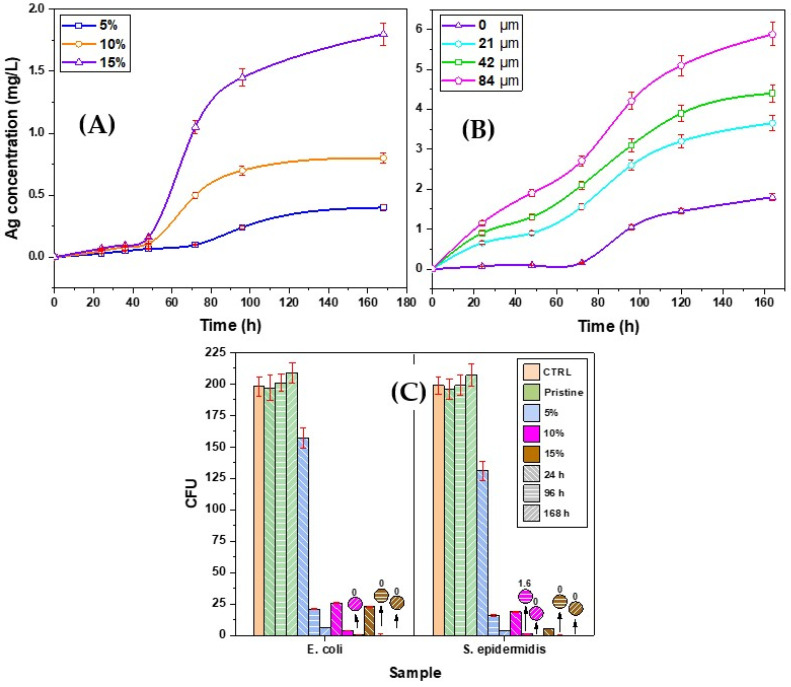
Concentrations of silver ions released into the water as a function of the original concentrations of Ag in solid samples and total soaking time (**A**); concentration of the Ag^+^ released into water from samples of concentration 15% as a function of the microstructure height and the total period of soaking 0: no microstructure (flat surface), 21, 42 and 84 μm (**B**); the number of colony-forming units (CFU) of *E. coli* and *S. epidermidis* (**C**).

AS measurement showed that the concentration of released Ag was proportional to the original concentrations of AgNPs in the solid samples and also showed the release acceleration with time (see Figure 5A).

Similar results were obtained for the study of surface microstructures with increasing height and in turn with increasing total surface and wider contact between the material and the deionized water. The AAS results presented in Figure 5B confirmed an increase in the concentration of the released Ag with increasing height of the microstructure. This offers a possibility to control the amount of the released ions without changing the shape or size of the total specimen. This might be of significant importance for the compatibility between the available space and location of application and the required antibacterial effect depending on the concentration of the released ions.

Subsequent antibacterial tests showed that the release of Ag^+^ ions from the samples in the environment was at sufficient concentration to have an antibacterial effect. This ability increased significantly with the concentration of the Ag^+^ ions into the solutions and the period of soaking. As it could be expected, higher concentrations of the modified AgNPs in the solid samples and/or microstructure with larger surface area provide better antibacterial effects. The antibacterial effects of the prepared materials were more pronounced for *S. epidermidis* in comparison with *E. coli* (Figure 5C).

**Figure 6:**
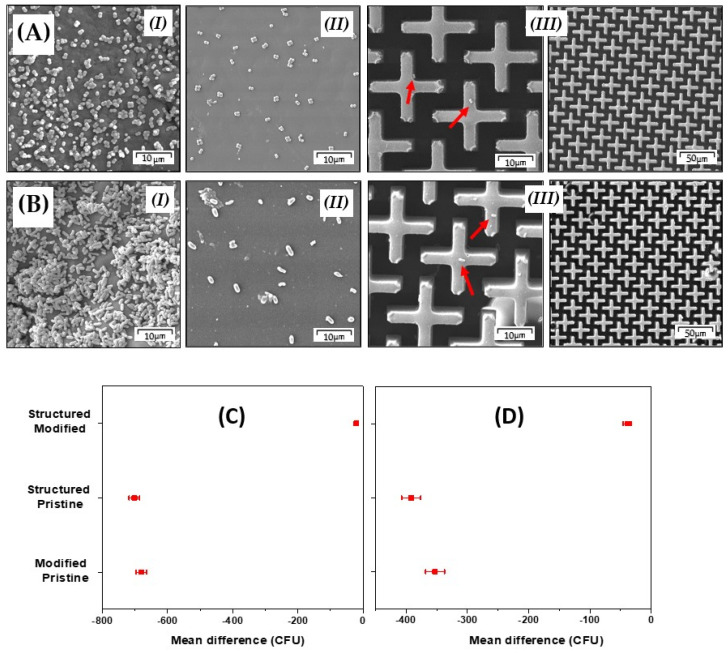
Results of antifouling tests performed by SEM for bacterial strains of *S. epidermidis* (**A**) and *E. coli* (**B**); results of analysis of variance one way (ANOVA) between groups for *S. epidermidis* (**C**) and *E. coli* (**D**).

The antifouling tests by SEM showed minimal bacteria adhesion on the structured surface in comparison with the “flat” one. The cultivation of bacteria was limited and the colonies of bacteria were small and separated (see Figure 6). Also, interpretation of ANOVA analysis results shows that the mean difference between the structured samples and pristine samples was significant at the level of 0.05 for both bacterial strains (see Figure 6C,D).

These results show a high antibacterial/antifouling effect, and perhaps unlike many previous methods [28] in preparation of antibacterial printable resin, it preserved the original properties of the resin.

## 4. Conclusions

In this study, an alternative method for modifying the SDL-compatible resin with AgNPs was described. Commercially available AgNPs were grafted with MMA in situ using 4-aminostyrene. The success of this modification was confirmed by FTIR spectroscopy and changes in the nanoparticle behavior were observed by TEM. Adding the modified AgNPs did not affect the physical characterization of the resulting liquid in terms of its peak absorption and viscosity. After the samples solidified it was proved that the distribution of AgNPs was homogenous and that the addition of the AgNPs did not affect the mechanical properties of the samples, as proved by SEM-EDX and indication tests. The AAS test confirmed that the samples are capable of releasing Ag^+^, thus they had an antibacterial effect against *S. epidermidis* and *E. coli*. The ductility of the resin remained unaffected by adding AgNPs which made it possible to print antifouling microstructures and microstructures for controlling the release of Ag^+^ while maintaining the original sample dimensions and the concentration of AgNPs in solid samples unchanged. This facile approach shows promising results and preserved all the latent potential of the resin, allowing more versatile applications which may advance research on antibacterial materials.

## Figures and Tables

**Table 1:** Mechanical characterization of different samples.

Sample	H (MPa)	E (GPa)
**Pristine**	198.3 ± 8.4	4.0 ± 0.2
**Modified**	185.0 ± 17.3	3.5 ± 0.2
**Modified after 1 week**	201.9 ± 14.5	3.8 ± 0.1
**Modified after 1 month**	194.9 ± 16.9	3.8 ± 0.2

H—Hardness, E—Plane strain modulus of elasticity.

## Data Availability

All the data will be available to the readers.
